# Protective Effects of Opuntiol on Lipopolysaccharide-Induced Acute Kidney Injury in Mice

**DOI:** 10.33549/physiolres.935639

**Published:** 2026-02-01

**Authors:** Yuan LI, Chunhui YANG, Mengxin LIU, Chunyan LI, Xun LI, Lingqiang KONG

**Affiliations:** 1 Nephrology Department, Northwest University First Hospital, Xi’an, China

**Keywords:** Opuntiol, Acute kidney injury, Lipopolysaccharide, Oxidative stress, Inflammation, Apoptosis

## Abstract

This study investigates the protective effect of opuntiol, a naturally occurring flavonoid, against lipopolysaccharide (LPS)-induced acute kidney injury in mice. Acute kidney injury (AKI) is a serious clinical complication characterized by inflammation, oxidative stress, and apoptosis, often resulting in high morbidity and mortality. Male mice were divided into six groups and administered opuntiol (25, 50 and 100 mg/kg b. wt.) intraperitoneally prior to LPS (10 mg/kg b. wt.) administration. The most effective dose was 50 mg/kg b. wt., as indicated in the dose-finding study. Kidney function markers (urea, creatinine, blood urea nitrogen (BUN), uric acid), antioxidant enzymes superoxide dismutase (SOD), catalase (CAT), glutathione peroxidase (GPx), reduced glutathione (GSH), inflammatory cytokines tumor necrosis factor alpha (TNF-α), interleukin-6 (IL-6), Nuclear factor kappa-light-chain-enhancer of activated B cells (NF-κB), cyclooxygenase-2 (COX-2), and gene expression levels (pro-inflammatory, apoptotic, and antioxidant genes) were analyzed using biochemical assays and qRT-PCR. Opuntiol significantly reduced elevated levels of serum urea, creatinine, BUN, and uric acid compared to the LPS group. Further, opuntiol restored antioxidant enzyme activities and MDA levels were significantly decreased. Opuntiol also downregulated inflammatory markers and gene expressions (TNF-α, NF-κB, TLR4, Bax, Caspase-3, etc.) while upregulating anti-apoptotic (Bcl-2) and antioxidant (Nrf-2) genes. Opuntiol offers significant protection against LPS-induced AKI by mitigating oxidative damage, inflammation, and apoptotic signaling. It enhances renal function and promotes antioxidant defense. These findings support the therapeutic potential of opuntiol as a novel nephroprotective agent in managing sepsis-associated kidney injury and encourage further preclinical and clinical investigations.

## Introduction

Sepsis-induced acute kidney injury (SI-AKI) is a complex, multifactorial disorder marked by excessive inflammation, systemic endothelial dysfunction, and pathological changes in renal resident cells that can accelerate kidney disease progression and aging [1]. Lipopolysaccharide (LPS), a major structural component of the outer membrane of Gram-negative bacteria, plays a central role in the pathogenesis of sepsis. LPS is detected by Toll-like receptor 4 (TLR4) [2], an innate immune receptor expressed on both immune cells and renal resident cells. Activation of TLR4 signaling by LPS initiates a strong inflammatory cascade, resulting in the release of multiple pro-inflammatory mediators [3,4].

Excessive production of inflammatory mediators, including tumor necrosis factor-α (TNF-α), nuclear factor-κB (NF-κB), monocyte chemoattractant protein-1 (MCP-1), interleukin (IL)-6, and IL-1β, plays a pivotal role in the pathogenesis of sepsis-associated kidney injury [5]. Elevated levels of these cytokines and chemokines promote the recruitment and infiltration of immune cells such as macrophages and neutrophils into renal tissue, where they release reactive oxygen species that exacerbate tissue damage [6,7]. Therefore, targeting pro-inflammatory mediators represents a promising therapeutic strategy for attenuating kidney inflammation and injury [8].

Furthermore, apoptosis or programmed cell death is a hallmark feature of LPS-induced AKI. It occurs primarily in renal tubular epithelial cells and is mediated through both intrinsic (mitochondrial) and extrinsic (death receptor) pathways [9]. The intrinsic pathway is primarily triggered by mitochondrial dysfunction and oxidative stress, leading to the release of cytochrome c into the cytoplasm. This promotes the activation of caspase-9, which subsequently activates the effector caspase-3, resulting in DNA fragmentation and cell death [10]. LPS exposure also alters the balance of Bcl-2 family proteins, favoring pro-apoptotic members such as Bax over the anti-apoptotic protein Bcl-2, thereby amplifying mitochondrial permeability and accelerating apoptosis [11]. In parallel, the extrinsic apoptotic pathway is initiated through activation of death receptors such as Fas and TNF receptor-1, which recruit adaptor proteins and activate caspase-8. Activated caspase-8 can directly cleave and activate caspase-3 or cross-talk with the mitochondrial pathway to further enhance apoptosis [12]. The combined activation of these pathways results in extensive loss of renal tubular epithelial cells, contributing to tubular atrophy, brush border loss, and impaired regenerative capacity of the kidney during sepsis.

In this context, opuntiol, a naturally occurring flavonoid-like phytochemical isolated from *Opuntia* species (commonly known as prickly pear cactus), has emerged as a promising candidate with potential nephroprotective properties. *Opuntia ficus-indica*, in particular, is widely consumed in traditional diets and used in ethnomedicine for its anti-inflammatory, antioxidant, and hepatoprotective effects [13]. Opuntiol, one of its key bioactive components, has demonstrated radical scavenging activity, inhibition of pro-inflammatory cytokines, and modulation of NF-κB signa-ling in macrophages and liver injury models [14,15]. Despite its known pharmacological effects, the role of opuntiol in kidney injury, particularly in LPS-induced septic AKI, has not been previously elucidated. Considering its chemical profile and known bioactivity, opuntiol presents a strong therapeutic candidate for targeting multiple pathological pathways involved in S-AKI. This study, therefore, aimed to evaluate the protective effects of opuntiol in an LPS-induced mouse model of acute kidney injury, with specific focus on renal function markers, oxidative stress parameters, inflammatory cytokine levels, and apoptotic gene expression. We hypothesized that opuntiol would attenuate kidney injury by restoring redox balance, suppressing inflammatory responses, and inhibiting apoptosis.

## Materials and Methods

### Chemicals

Opuntiol was purchased from Chengdu Peter-like Biotechnology Co., Ltd. China (CAS: 2860-28-8). TNF-α, IL-2, NF-κB, COX-2, TLR-4, Bax, Bcl-2, Caspase-3, -9, cytochrome c, TLR-4 and Nrf-2 primers were obtained from Sigma-Aldrich, USA. Molecular biology reagents, including those for RNA isolation (TRIzol), reverse transcription, and quantitative PCR, were sourced from Invitrogen (USA). All other fine chemicals and solvents were purchased from Himedia, USA.

### Animals and experimental design

Adult male Swiss albino mice (8–10 weeks old, 25–30 g) were housed in polypropylene cages under controlled environmental conditions (22±2 °C, 50–60 % humidity, 12-hour light/dark cycle) with *ad libitum* access to water and a standard pellet diet. All experimental procedures were conducted in accordance with the CPCSEA guidelines and approved by the Institutional Animal Ethics Committee (IAEC/Approval No.: 2024KY-087, Northwest University First Hospital).

Mice were randomly divided into six groups (n=6 each):

Group I – Control (0.9 % saline)
Group II – Opuntiol (100 mg/kg b. wt.)
Group III – LPS (10 mg/kg b. wt.)
Group IV – LPS + Opuntiol (25 mg/kg b. wt.)
Group V – LPS + Opuntiol (50 mg/kg b. wt.)
Group VI – LPS + Opuntiol (100 mg/kg b. wt.)

Opuntiol was administered intraperitoneally 1 h prior to LPS injection (also intraperitoneal). Mice were sacrificed 24 h after LPS injection under pentobarbital anesthesia (60 mg/kg i.p.). Blood and kidney tissues were collected for analysis.

### Serum biochemistry

Blood was collected by retro-orbital puncture and allowed to clot. Serum was separated by centrifugation at 3000 rpm for 10 min and stored at −20 °C. Serum urea, creatinine, uric acid, and blood urea nitrogen (BUN) levels were determined using diagnostic kits (ERBA Diagnostics, India) and read using an automated clinical chemistry analyzer (Erba Chem 5 Plus V2, Transasia Bio-Medicals Ltd). These parameters reflect renal filtration efficiency and glomerular integrity [16].

### Antioxidant and oxidative stress assays

Kidney tissues were homogenized in cold phosphate-buffered saline (pH 7.4) using a Polytron PT 1200E homogenizer (Kinematica, Switzerland). The homogenate was centrifuged at 10000 rpm for 15 min at 4 °C and the supernatant used for biochemical assays. Lipid peroxidation was quantified by estimating malondialdehyde (MDA) levels using the thiobarbituric acid reactive substances (TBARS) assay. 0.5 ml of tissue homogenate was diluted with 0.5 ml double distilled water and mixed well, and then 2.0 ml of TBA-TCA-HCl reagent was added. The mixture was kept in a boiling water bath for 15 min, after cooling, the tubes were centrifuged at 1000× g for 10 min and the absorbance of supernatant was measured at 535 nm [17].

Superoxide dismutase (SOD) activity was assayed by the prescribed method [18]. First, 0.5 ml tissue homogenate was diluted to 1.0 ml with water followed by addition of 2.5 ml of ethanol and 1.5 ml of chloroform (chilled reagents were added). This mixture was shaken for 90 s at 4 °C and then centrifuged. The enzyme activity in the supernatant was determined as follows. The assay mixture contained 1.2 ml of sodium pyrophosphate buffer, 0.1 ml of phenazine methosulfate, and 0.3 ml of nitroblue tetrazolium and appropriately diluted enzyme preparation in a total volume of 3.0 ml. The addition of 0.2 ml NADH started the reaction. After incubation at 30 °C for 90 s, the reaction was stopped by adding 1.0 ml glacial acetic acid. The reaction mixture was stirred vigorously and shaken with 4.0 ml n-butanol. The mixture was allowed to stand for 10 min; centrifuged and the n-butanol layer was separated. The color density of the chromogen in n-butanol was measured in at 510 nm.

Catalase (CAT) activity was assayed by Sinha *et al.* [19]. First, 0.9 ml of phosphate buffer, 0.1 ml of tissue homogenate, and 0.4 ml of hydrogen peroxide were added. The reaction was arrested after 15, 30, 45, and 60 s incubation at 37 °C by adding 2.0 ml of dichromate-acetic acid mixture. The tubes were kept in a boiling water bath for 10 min, cooled, and the color developed was read at 620 nm.

Glutathione peroxidase (GPx) activity was determined by the oxidation of reduced glutathione using cumene hydroperoxide [20]. 0.2 ml of tris buffer, 0.2 ml of EDTA, 0.1 ml of sodium azide, and 0.5 ml of tissue homogenate were added. Then, 0.2 ml of GSH followed by 0.1 ml of H_2_O_2_ was added to the mixture. The contents were mixed well and incubated at 37 °C for 10 min, along with a control containing all reagents except homogenate. After 10 min, the reaction was arrested by adding 0.5 ml of 10 % TCA. The tubes were centrifuged and the supernatant was assayed for GSH by the method of Ellman.

GSH was estimated by Ellman method [21]. In all the experimental tubes 0.5 ml of tissue homogenate and 2.0 ml of 5 % TCA were added and allowed to stand for 5 min. After centrifugation, 2.0 ml supernatant was taken and 1.0 ml of Ellman’s reagent and 4.0 ml of 0.3 M disodium hydrogen phosphate were added. The yellow color developed was read at 412 nm. All enzyme activities were normalized to the protein content determined by the Bradford method using bovine serum albumin as a standard.

### Estimation of serum cytokines

Serum levels of TNF-α, IL-6, NF-κB, and COX-2 were measured using ELISA kits as per manufacturer’s instructions (Invitrogen, USA). In the protocol, 50 μl of the sample and 50 μl of an antibody cocktail were added to the experimental wells and incubated at 37 °C for 1 h. The wells were then washed with 100 μl of 3,3′,5,5′-Tetramethylbenzidine substrates and incubated for 10 min. Finally, 100 μl of stop solution was added, and the color development was read at 450 nm using a microplate reader (BioTek ELx800, USA). The results were expressed as pg/ml based on standard calibration curves [22].

### Gene expression analysis by qRT-PCR

Total RNA was extracted from frozen kidney tissue using TRIzol reagent (Invitrogen, Thermo Fisher Scientific, USA). Kidney tissue (30 mg) was homogenized after addition of appropriate buffer (RLT) supplied in the kit and the tissue lysate was centrifuged at maximum speed (15000 rpm) for 3 min in a micro-centrifuge. After centrifugation, the supernatant was carefully transferred to a new Eppendorf tube. Equal volume of 70 % ethanol was then added to the supernatant and mixed well. From this, 700 μl along with the precipitate formed was transferred to RNeasy mini spin column and was centrifuged at 11000 rpm for 15 s. After addition of RW1 and RPE buffers the centrifugation process was repeated thrice and the elution step was performed by transferring the RNeasy column to a new 1.5 ml collection tube. 30–50 μl of RNase free water was then added directly onto the tube containing the membrane and was centrifuged at 11000 rpm for 1 min. The elution step was repeated to obtain higher yield of RNA (30–50 μg). Finally, the RNeasy spin column was discarded and the tube containing RNA was stored at −80 °C. RNA quantity and purity were assessed using a NanoDrop 2000 spectrophotometer (Thermo Fisher Scientific, USA). One microgram of RNA was reverse transcribed to cDNA using iScript™ cDNA synthesis kit (Bio-Rad, USA). Quantitative real-time PCR was performed using SYBR Green PCR Master Mix (Thermo Fisher Scientific, USA) in a StepOnePlus™ Real-Time PCR System (Applied Biosystems, USA). Gene expression levels of inflammatory (TNF-α, IL-2, NF-κB, COX-2, TLR-4), apoptotic (Bax, Bcl-2, Caspase-3, Caspase-9, Cytochrome-c), and antioxidant response gene (Nrf-2) were normalized against GAPDH. Primers (Table 1) were designed using Primer3 software and validated through melting curve analysis. Data were analyzed using the 2^^−ΔΔCt^ method [23].

### Statistical analysis

All experimental values were expressed as means ± standard deviation (SD). Statistical comparisons were made using one-way analysis of variance (ANOVA) followed by Duncan’s Multiple Range Test (DMRT) using SPSS software version 25.0 (IBM Corp., USA). A p-value <0.05 was considered statistically significant.

## Results

### Dose fixation study

To determine the most effective nephroprotective dose of opuntiol, mice were pre-treated with 25, 50, or 100 mg/kg body weight (b. wt.) opuntiol before LPS administration. As shown in Table 2, LPS (10 mg/kg) induced a significant elevation (p<0.05) in serum levels of urea (64.45±7.171 mg/dl), uric acid (08.10±0.70 mg/dl), creatinine (2.89±0.38 mg/dl), and BUN (92.82±9.35 mg/dl) compared to control mice. Among the tested doses, 50 mg/kg opuntiol significantly (p<0.05) reduced these levels more effectively than the 25 mg/kg and 100 mg/kg doses, restoring urea (35.26±3.69 mg/dl), uric acid (03.12±0.29 mg/dl), creatinine (01.04±0.14 mg/dl), and BUN (48.14± 5.80 mg/dl). The 100 mg/kg dose, though effective, was slightly less protective than the 50 mg/kg dose, suggesting a non-linear dose-response relationship. Therefore, 50 mg/kg b. wt. was selected as the optimal dose for subsequent experiments.

### Opuntiol improves renal function markers

As seen in Table 3, LPS exposure significantly (p<0.05) impaired renal function, increasing serum levels of urea, uric acid, creatinine, and BUN compared to the control group. Treatment with opuntiol (50 mg/kg b.wt.) led to a significant (p<0.05) reduction in these values – restoring them closer to physiological levels. These statistically significant differences indicate that opuntiol confers substantial protection against LPS-induced renal dysfunction.

### Opuntiol attenuates oxidative stress

LPS-induced oxidative stress was characterized by a significant (p<0.05) elevation in MDA levels and a reduction in antioxidant defenses, as summarized in Table 4. Treatment with opuntiol significantly reversed these changes, increasing SOD, CAT, GPx, and GSH activities while reducing MDA to near control levels (p<0.05). These results clearly demonstrate that opuntiol restores redox homeostasis and mitigates lipid peroxidation in LPS-challenged mice.

### Opuntiol suppresses inflammatory cytokine production

The LPS group exhibited a significant (p<0.05) increase in systemic inflammation, evidenced by elevated serum levels of TNF-α, IL-6, NF-κB, and COX-2 as shown in Table 5. Opuntiol significantly (p<0.05) suppressed these inflammatory markers, affirming its role as a potent anti-inflammatory agent in septic AKI.

### Opuntiol modulates inflammatory and apoptotic gene expression

**Fig. 1:**
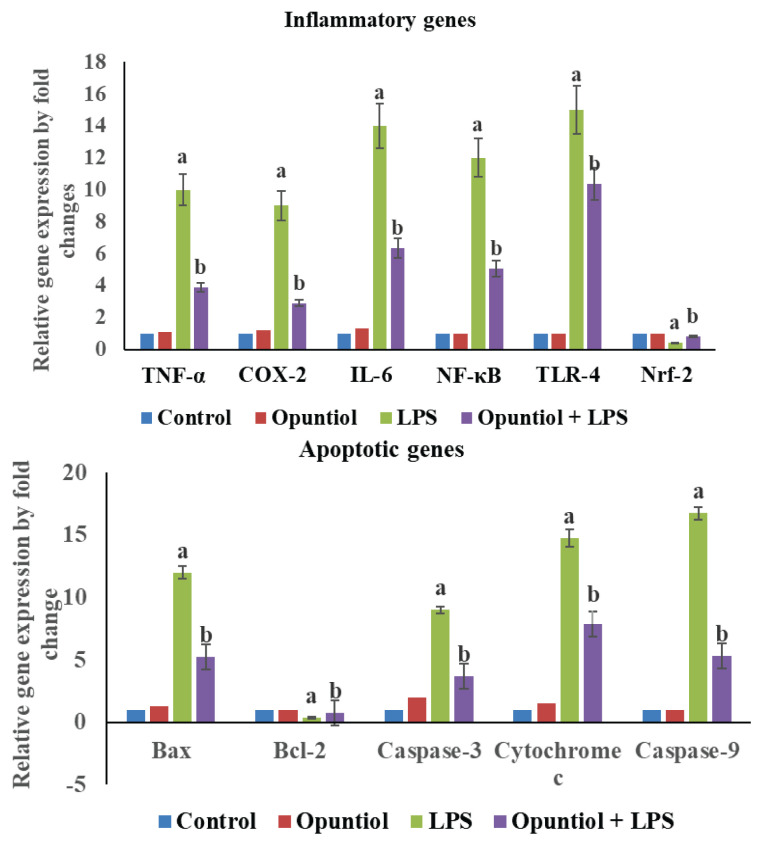
(**A**) Effect of opuntiol on LPS-induced inflammation markers gene expressions in the kidney of control and experimental mice. (**B**) Effect of opuntiol on LPS-induced apoptotic markers gene expressions in the kidney of control and experimental mice. Histogram depicts the quantitation of three independent experiments (means ± S.D). The TNF-α, IL-2, COX-2, NF-κB, TLR-4, Bax, Bcl-2, Caspase-3, Caspase-9, cytochrome-c and Nrf-2 genes expression levels were normalized with the expression level of the GAPDH mRNA in each sample.

Gene expression analysis *via* qRT-PCR revealed that LPS significantly (p<0.05) upregulated inflammatory genes like TNF-α, COX-2, IL-6, NF-κB. TLR-4 and pro-apoptotic markers Bax, Caspase-3, −9 and cytochrome c, while downregulating anti-apoptotic gene BCl-2 and antioxidant gene Nrf-2 in renal tissues (Fig. 1A, B). Opuntiol treatment reversed these effects, suggesting suppression of inflammation, apoptosis and enhanced anti-apoptotic and antioxidant gene when compared to LPS-induced mice.

## Discussion

AKI remains a major clinical challenge, contributing significantly to morbidity and mortality in critically ill patients. The pathogenesis of AKI is multifactorial, involving hemodynamic alterations, systemic inflammation, oxidative stress, and apoptosis. In this context, this present study evaluated the nephroprotective efficacy of opuntiol, a naturally occurring flavonoid derived from *Opuntia ficus-indica*, in an LPS-induced AKI mouse model. LPS administration significantly impaired renal function, as evidenced by elevated serum levels of creatinine, BUN, and uric acid compared to the control group, indicating acute kidney injury. These changes are consistent with earlier reports that endotoxemia provokes renal dysfunction by triggering systemic inflammation and hemodynamic instability [24,25]. The present findings demonstrate that pre-treatment with opuntiol significantly ameliorates renal dysfunction caused by LPS challenge. This was evidenced by reduced levels of serum urea, uric acid, creatinine, and BUN. These results are consistent with earlier studies involving plant-derived compounds such as resveratrol, curcumin, and carnosic acid [26]. However, the present study is the first to report such effects for opuntiol in the context of renal inflammation and injury.

It has been observed that opuntiol pretreatment restored oxidative balance in LPS-challenged kidneys by reducing lipid peroxidation and enhancing antioxidant enzyme activities. These changes signify a restoration of redox homeostasis, crucial in preventing ROS-mediated tubular damage. Compared to flavonoids like quercetin and luteolin, opuntiol showed protective effects at a moderate dose, indicating strong intrinsic antioxidant capacity [27].

LPS administration markedly increased renal MDA level, reflecting enhanced lipid peroxidation and oxidative tissue damage. Elevated MDA is a well-established indicator of oxidative stress and is closely linked with membrane injury and loss of cellular integrity in septic AKI [28]. Concomitantly, the activities of key endogenous antioxidants, including superoxide dismutase (SOD), catalase (CAT), glutathione peroxidase (GPx), and reduced glutathione (GSH), were significantly reduced in LPS-treated mice compared to the control group. This depletion of enzymatic and non-enzymatic antioxidant defenses suggests that overwhelming ROS generation during endotoxemia exceeded the renal antioxidant buffering capacity, thereby aggravating tissue injury. Opuntiol administration antioxidant action can be attributed to its polyphenolic structure, which allows direct neutralization of reactive oxygen species and modulation of redox-sensitive transcription factors such as NF-κB and Nrf2. By reinforcing antioxidant defenses and reducing lipid peroxidation, opuntiol protects renal tissue against oxidative stress-mediated apoptosis and functional impairment in septic AKI [29].

The current study demonstrates that LPS challenge in mice significantly upregulated key pro-inflammatory genes (TNF-α, COX-2, IL-6, NF-κN, TLR-4) and pro-apoptotic markers (Bax, Caspase-3, Caspase-9, Cytochrome c), while concomitantly downregulating the anti-apoptotic gene Bcl-2 and the antioxidant transcription factor Nrf-2 in renal tissues. These transcriptional alterations are consistent with previous reports showing that endotoxin-induced AKI is characterized by TLR4-mediated activation of NF-κB signaling, leading to enhanced transcription of inflammatory cytokines and mediators, oxidative stress, and apoptosis in renal tubular epithelial cells [30,31].

Opuntiol treatment significantly counteracted these deleterious processes. Opuntiol is known to possess potent radical scavenging activity and the ability to stabilize redox homeostasis [32]. In our study, opuntiol administration restored antioxidant enzyme activities and reduced lipid peroxidation, suggesting direct quenching of reactive oxygen species (ROS) and preservation of mitochondrial integrity. This antioxidant effect likely underlies the observed decrease in cytochrome c release and caspase activation. Furthermore, the downregulation of NF-κB signaling by opuntiol indicates that it interrupts the inflammatory cascade at a central transcriptional hub, thereby lowering TNF-α and IL-6 production and preventing sustained immune-mediated injury. These dual antioxidant and anti-inflammatory effects position opuntiol as a superior candidate compared to conventional single-target therapies.

The anti-apoptotic action of opuntiol was also evident from the restoration of the Bax/Bcl-2 ratio and suppression of caspase-3 activity. Preservation of Bcl-2, a key mitochondrial stabilizer, suggests that opuntiol maintains mitochondrial integrity and prevents the amplification of intrinsic apoptosis. Similar anti-apoptotic effects have been reported with other plant-derived compounds, such as resveratrol and quercetin [33,27], but opuntiol’s combined impact on redox balance, cytokine suppression, and apoptotic signaling highlights its multitargeted pharmacology. Unlike synthetic agents that often focus on a single molecular pathway, phytochemicals such as opuntiol may offer broader protective effects with favorable safety profiles.

This study is the first to report opuntiol’s renoprotective efficacy in a validated model of AKI. Given its origin from a widely consumed cactus plant, opuntiol is pharmacologically safe and ethnomedicinally relevant. Its effectiveness at a moderate dose and ability to act on multiple pathological axes highlight its therapeutic promise.

## Conclusions

This study demonstrates that opuntiol exerts significant protective effects against LPS-induced acute kidney injury in mice by improving renal function, restoring antioxidant defenses, suppressing pro-inflammatory cytokines, and modulating apoptotic signaling pathways. These findings highlight opuntiol’s potential as a multitargeted, plant-derived therapeutic candidate for the management of sepsis-associated kidney injury. Further studies are warranted to explore its clinical applicability and mechanistic pathways in greater detail.

## Figures and Tables

**Table 1:** Primer sequences.

*Gene*	Forward Primer (5′→3′)	Reverse Primer (5′→3′)
*TNF-α*	CAGGCGGTGCCTATGTCTC	CGATCACCCCGAAGTTCAGTAG
*IL-6*	CTGAGCAGGATGGAGAATTACAGG	GTCCAAGTTCATGTCCACTTCTGT
*NF-κB p65*	GGGAAGGCAGCAAAGTCTGG	TCATCCTCATCTTGTTTCCCCT
*COX-2*	TTCAAATGAGATTGTGGGAAAATTGCT	GATACACCTCTCCACCAATGCT
*TLR4*	TGAGCAGAGGTGGAATGCTG	CTGAGCAGGGTCTTCTCCAC
*Bax*	AGCTGAGCGAGTGTCTCAAG	CAGTTGAAGTTGCCGTCAGA
*Bcl-2*	ATCGCCCTGTGGATGACTGAGTAC	AGAGACAGCCAGGAGAAATCAAAC
*Caspase-3*	TGTCATCTCGCTCTGGTACG	AAATGACCCCTTCATCACCA
*Caspase-9*	GCAGCCACGCTGTGATGTC	GTCGGCCATCTCATCAAACAT
*Cytochrome-c*	GGCATCACGGTGTACTTTGC	CGGTAGATGCCCTTTTCCTT
*Nrf-2*	AATAGCTGAGCCCAGTGTGC	TCTGCTTGTTTGGGAATGTG
*GAPDH*	AGGTCGGTGTGAACGGATTTG	TGTAGACCATGTAGTTGAGGTCA

**Table 2:** Effect of opuntiol on kidney function markers in the serum (dose-fixation study) of control and LPS-induced mice.

*Groups*	Urea (mg/dl)	Uric acid (mg/dl)	Creatinine (mg/dl)	BUN (mg/dl)
*Control*	31.82 ± 4.15^a^	01.03 ± 0.10^a^	0.90 ± 0.12^a^	36.30 ± 3.96^a^
*Opuntiol control (100 mg/kg b. wt.)*	31.62 ± 3.92^a^	01.05 ± 0.11^a^	0.93 ± 0.14^a^	37.19 ± 4.15^a^
*LPS control (10 mg/kg b. wt.)*	64.45 ± 7.17^b^	08.10 ± 0.70^b^	2.89 ± 0.38^b^	92.82 ± 9.35^b^
*Opuntiol (25 mg/kg b. wt.) + LPS (10 mg/kg b. wt.)*	50.24 ± 4.92^c^	06.71 ± 0.59^c^	1.92 ± 0.25^c^	72.48 ± 8.83^c^
*Opuntiol (50 mg/kg b. wt.) + LPS (10 mg/kg b. wt.)*	35.26 ± 3.69^d^	03.12 ± 0.29^d^	1.04 ± 0.14^d^	48.14 ± 5.80^d^
*Opuntiol (100 mg/kg b. wt.) + LPS (10 mg/kg b. wt.)*	42.70 ± 4.46^e^	04.19± 0.53^e^	1.41 ± 0.16^e^	59.01 ± 6.32^e^

Values are expressed as means ± S.D for six mice in each group. Values not sharing a common marking superscript (^a, b, c^) are significantly different at p-value ≤0.05 (DMRT).

**Table 3:** Effect of opuntiol on kidney function markers in the serum of control and LPS-induced mice.

*Parameters*	Control	Opuntiol	LPS	LPS + Opuntiol
*Urea (mg/dl)*	28.57 ± 2.91^a^	27.50 ± 3.23^a^	58.95 ± 5.71^b^	34.51 ± 4.79^c^
*Uric acid (mg/dl)*	1.19 ± 0.12^a^	1.15 ± 0.18^a^	7.82 ± 0.34^b^	3.25 ± 0.31^c^
*Creatinine (mg/dl)*	0.75 ± 0.07^a^	0.73 ± 0.09^a^	2.08 ± 0.31^b^	1.04 ± 0.12^c^
*BUN (mg/dl)*	30.36 ± 3.48^a^	32.96 ± 4.14^a^	81.24 ± 7.92^b^	48.95 ± 5.83^c^

Values are expressed as means ± S.D for six mice in each group. Values not sharing a common marking superscript (^a, b, c^) are significantly different at p-value <0.05 (DMRT).

**Table 4:** Effect of opuntiol on LPS-induced antioxidant status in kidney tissue of control and experimental rats.

*Parameters*	Control	Opuntiol	LPS	LPS + Opuntiol
*MDA (MDA (mmol/100 g wet tissue)*	7.58 ± 0.79^a^	7.63 ± 0.95^a^	13.93 ± 0.79^b^	9.02 ± 0.83^c^
*SOD (U* *^1^* */mg protein)*	7.12 ± 0.91^a^	7.02 ± 0.61^a^	3.67 ± 0.24^b^	6.34 ± 0.83^c^
*CAT (U* *^2^* */mg protein)*	48.76 ± 5.92^a^	49.61 ± 5.32^a^	19.73 ± 1.38^b^	36.21 ± 7.30^c^
*GPx (U* *^3^* */mg protein)*	6.33 ± 0.71^a^	6.29 ± 0.61^a^	4.01 ± 0.52^b^	6.10 ± 0.59^a^
*GSH (mg/100 g wet tissue)*	5.07 ± 0.92^a^	5.11 ± 0.83^a^	2.89 ± 0.40^b^	4.73 ± 0.56^c^

U^1^ = enzyme concentration required to inhibit the chromogen produced by 50 % in one minute under standard conditions. U^2^ = μmole of H_2_O_2_ consumed/minute. U^3^ = μg of GSH utilized/minute. Values are expressed as means ± S.D for mice rats in each group. Values not sharing a common marking superscript (^a, b, c^) are significantly different at p-value <0.05 (DMRT).

**Table 5:** Effect of Opuntiol on LPS-induced inflammatory cytokines in the serum of control and experimental rats.

*Parameters*	Control	Opuntiol	LPS	LPS + Opuntiol
*TNF-α (pg/ml)*	49.55± 5.08^a^	49.33± 5.82^a^	97.98 ± 7.54^b^	58.59 ± 7.80^c^
*IL-6 (pg/ml)*	80.68 ± 7.80^a^	80.74 ± 9.35^a^	142.73 ± 11.57^b^	98.62 ± 9.31^c^
*NF-κB (pg/ml)*	24.09 ± 1.94^a^	25.02 ± 3.38^a^	73.63 ± 8.53^b^	32.94 ± 4.26^c^
*COX-2 (pg/ml)*	24.84 ± 03.58^a^	24.91 ± 3.95^a^	64.95 ± 8.10^b^	37.73 ± 4.39^c^

Values are expressed as means ± S.D for six mice in each group.

b Significantly different from the control group (p<0.05).

c Significantly different from the LPS group (p<0.05).

Values not sharing a common marking superscript (^a,b,c^) are significantly different at p-value <0.05 (DMRT).

## Abbreviations

AKI: Acute kidney injury
Bax: Bcl-2-associated x protein
Bcl-2: B-cell lymphoma 2
BUN: Blood urea nitrogen
CAT: Catalase
COX-2: Cyclooxygenase-2
GAPDH: Glyceraldehyde-3-Phosphate Dehydrogenase GPx, Glutathione peroxidase
GSH: Reduced glutathione
IL-6: Interleukin-6
LPS: Lipopolysaccharide
MDA: Malondialdehyde
NF-κB: Nuclear factor kappa-light-chain-enhancer of activated b cells
Nrf-2: Nuclear factor erythroid 2-related factor 2
SOD: Superoxide dismutase
TLR4: Toll-like receptor 4
TNF-α: Tumor necrosis factor alpha
